# Knock-Down of *Histidyl-tRNA Synthetase* Causes Cell Cycle Arrest and Apoptosis of Neuronal Progenitor Cells *in vivo*

**DOI:** 10.3389/fcell.2019.00067

**Published:** 2019-04-26

**Authors:** Ashley Waldron, Claire Wilcox, Christopher Francklyn, Alicia Ebert

**Affiliations:** ^1^Department of Biology, The University of Vermont, Burlington, VT, United States; ^2^Department of Biochemistry, The University of Vermont, Burlington, VT, United States

**Keywords:** aminoacyl-tRNA synthetase, histidyl-tRNA synthetase, HARS, retina, Cyclin D1, CCND1, proliferation, apoptosis

## Abstract

Histidyl-tRNA Synthetase (HARS) is a member of the aminoacyl-tRNA synthetase family, which attach amino acids to their associated tRNA molecules. This reaction is a crucial step in protein synthesis that must be carried out in every cell of an organism. However, a number of tissue-specific, human genetic disorders have been associated with mutations in the genes for aminoacyl-tRNA synthetases, including HARS. These associations indicate that, while we know a great deal about the molecular and biochemical properties of this enzyme, we still do not fully understand how these proteins function in the context of an entire organism. To this end, we set out to knock-down HARS expression in the zebrafish and characterize the developmental consequences. Through our work we show that some tissues, particularly the nervous system, are more sensitive to HARS loss than others and we reveal a link between HARS and the proliferation and survival of neuronal progenitors during development.

## Introduction

Translation of mRNA molecules into protein is a fundamental cellular process. One family of translation proteins are the aminoacyl-tRNA synthetases (ARS), which are responsible for attaching amino acids to their cognate tRNA molecules (Ibba and Soll, 2000). For each proteinergic amino acid, there is a dedicated ARS; for example, histidyl-tRNA synthetase (HARS) attaches histidine to its corresponding tRNA molecules. Therefore, these enzymes are critical for maintaining the fidelity of the genetic code and for organismal function. Over the past decade or so, a number of human disorders have been connected to mutations in genes for ARS (Antonellis and Green, 2008; Meyer-Schuman and Antonellis, 2017). Despite the ubiquitous nature of ARS, many of these diseases effect specific tissues, illustrating how much we still need to learn about these proteins’ influence over organismal form and function.

The system most frequently effected in aminoacyl-tRNA synthetase related diseases is the nervous system. Dominant mutations in several synthetases have been shown to cause the peripheral neuropathy Charcot-Marie-Tooth (CMT), while many recessive mutations have been associated with a range of central nervous system defects from microcephaly to hearing loss (Meyer-Schuman and Antonellis, 2017). For example, HARS has been associated with both CMT and a combined deafness-blindness disorder making it an appealing synthetase to investigate (Puffenberger et al., 2012; Vester et al., 2013; Brozkova et al., 2015). The association between synthetases and neurological diseases suggest that these enzymes may be playing a role that is particularly important for nervous system development and maintenance.

To date, there have been a number of studies on ARS function in animal models. One of the first models was a mouse strain with a mutation in alanyl-tRNA synthetase (AARS) that exhibited severe loss of cerebellar Purkinje neurons within the first year of life (Lee et al., 2006). Around the same time, another mouse strain that showed signs of peripheral neuropathy was found that had a mutation in glycyl-tRNA synthetase (GARS), reminiscent of the human CMT patients (Seburn et al., 2006). Interestingly, these mutations do not appear to be clear-cut loss of function alleles. However, this is not the case for most disease-associated mutations, and in fact many of the mutations do exhibit at least partial loss of aminoacylation function. For example, mutations in HARS have been shown to reduce aminoacylation activity by reducing stability or impairing substrate binding (Abbott et al., 2017a,b). Total loss of function would presumably result in early lethality, but there have been few studies on the influence partial loss of function has on the nervous system.

In this study, we set out to characterize the consequences of simply reducing expression of HARS in a vertebrate model system. Using zebrafish embryos we were able to knock down HARS expression and assess how development was disrupted *in vivo*. We focused on the impact that loss of HARS had on the retina as it is an accessible, highly ordered, relatively simple component of the central nervous system (London et al., 2013). Surprisingly, we found that the nervous system (as assessed by the retina) is particularly sensitive to a global reduction in HARS expression. It appears that the progenitor cells that give rise to all of the mature retinal cell types are the most severely affected by HARS knock-down. These neural progenitors exhibit cell cycle arrest and cell death in response to HARS knock down. Furthermore, we found that this effect can be rescued by overexpression of the cell cycle regulator Cyclin D1, suggesting that HARS is required for cell cycle progression upstream of Cyclin D1. Our results indicate that the proliferation of retinal progenitors is especially sensitive to levels of HARS and suggest that there are spatial differences in the demand for HARS throughout the organism.

## Materials and Methods

### Zebrafish Husbandry and Injection

Procedures were approved by the University of Vermont Institutional Animal Care and Use Committee Protocol Number: 14-053 and the University of Vermont Institutional Biosafety Committee Protocol Number: 14-024. Embryos were raised under standard conditions and staged as previously described (Kimmel et al., 1995; Westerfield, 2000). Strains used include: TL; *Tg(Rx3:GFP)* to label retinal progenitor cells (Rembold et al., 2006); *Tg(pou4f3:GFP)* to label sensory hair cells (Xiao et al., 2005); *Tg(ngn1:GFP)* to label sensory neurons (Blader et al., 2003); and *Tg(mnx1:mCherry)* to label motor neurons (provided by Christine Beattie, Ohio State University). Fertilized embryos were raised at 28.5 or 25°C and staged as previously described (Kimmel et al., 1995). In some cases, phenylthiourea was added to the embryo media at a concentration of 0.003% at 24 h post fertilization (hpf) in order to inhibit pigment formation.

Injections were performed at the 1-2 cell stage using an Eppendorf Femtojet 4i microinjector. A translation blocking *hars* morpholino (ATGGTGCTCCAGAAACACAGCCGAT), *p53* morpholino (Robu et al., 2007), and GeneTools Standard Control Oligo (GeneTools, Philomath, OR, United States) were injected at the amounts indicated in results. Human *HARS* and zebrafish *ccnd1* mRNA were injected at a dose of 200 pg.

### Cloning and *in vitro* Transcription

Total RNA was isolated from manually dechorionated embryos at 48 hpf using Trizol:chloroform (Invitrogen, Carlsbad, CA, United States), and used to synthesize first strand cDNA using Reverse Transcriptase (Applied Biosystems, Foster City, CA, United States) as described in the product manual. Table 1 contains the primers used to amplify *asns* (NM_201163), *gpt2* (NM_001098757), *eif4ebp1* (NM_199645), and *ccnd1* (NM_131025) using Q5 DNA Polymerase (New England Biolabs, Ipswich, MA). Q5 amplified products were cloned into pCR-Blunt-II TOPO using the Zero Blunt TOPO Cloning Kit (Invitrogen, Carlsbad, CA, United States) and sequence verified by the UVM Cancer Center Vermont Integrative Genomics Resource using M13 primers.

**Table 1:** Primer sequences used for PCR amplification.

	Forward	Reverse
*asns*	ATTTAGGTGACACTATAGGGTGTGTTCGCCTTCGCCTTCATCTT	TAATACGACTCACTATAGGGCATCTGGACTGTCCTCAGCA
*gpt2*	ATTTAGGTGACACTATAGATGACTATTCTGCGGCTGCT	TAATACGACTCACTATAGGGAAGGGTGGACACAGTCGAAC
*eif4ebp1*	ATTTAGGTGACACTATAGAGTCAGGCAATTCCAACCAC	TAATACGACTCACTATAGGGGCGACAGCATCAGTACAGGA
*ccnd1*	CTATTAAGCTTAGTTTTGTCAAGCGGAGAGC	CTATTTCTAGATTTCCCTCTTGTCCCATGAC
*Dr-hars*	ATTTAGGTGACACTATAGCAAAAGTGAGAAAGCGAGCA	TAAGACTCACTATAGGGTCAGGGATCATTGCATCGTA
*Hs-HARS*	TAATACGACTCACTATAGGG	CTGATCAGCGGGTTTAAT
*eif1α*	CGGTGACAACATGCTGGAGG	ACCAGTCTCCACACGACCCA

After sequencing, full length *ccnd1* mRNA was made by linearizing the pCR-Blunt-II TOPO-ccnd1 plasmid with XbaI (New England Biolabs, Ipswich, MA, United States). Linearized DNA was used as a template for *in vitro* transcription using the Sp6 mMessage mMachine Kit (Invitrogen, Carlsbad, CA, United States) as described in product manual. Transcripts were polyadenylated using a Poly(A) Tailing Kit (Invitrogen, Carlsbad, CA, United States) as described in the product manual.

The *ccnd1 in situ* probe was made by linearizing the pCR-Blunt-II-TOPO-ccnd1 plasmid with SacI (New England Biolabs, Ipswich, MA, United States). Zebrafish *asns*, *gpt2*, and *eif4ebp1 in situ* probes were made from EcoRI digested pCR-Blunt-II TOPO plasmids and transcribed using a T7 RNA polymerase (Affymetrix, Santa Clara, CA, United States) and DIG-labeling mix (Roche, Indianapolis, IN). Zebrafish *hars* (NM_001302262) *in situ* probes were made by PCR amplifying *hars* with the primers shown in Table 1 (*Dr-hars*) and transcribed using a T7 RNA polymerase as above.

The human *HARS* coding sequence (NM_002109) was gifted to us by Dr. Anthony Antonellis, University of Michigan. The primers used to amplify the coding sequence of *HARS* from the pcDNA vector are shown in Table 1 (*Hs-HARS*). The product was then transcribed using a T7 mMessage mMachine Kit (Invitrogen, Carlsbad, CA, United States) and polyadenylated as above.

### RT-PCR

Whole uninjected and *hars* KD embryos were collected at 48 hpf and RNA was isolated as above. For each group, equal amounts of RNA were used to generate cDNA as above. We used the same primers for *asns*, *gpt2*, and *eif4ebp1* as used to make the *in situ* probes (described above) and performed PCR using Q5 DNA Polymerase (New England Biolabs, Ipswich, MA, United States). As a loading control, we used the *eif1α* primers shown in Table 1. Equal volumes of each PCR were run on a 2% agarose gel stained with 1:10,000 SybrSafe (Invitrogen, Carlsbad, CA, United States). Gel images were captured using a Syngene GeneSys imaging system and ImageJ was used to measure mean gray values for densitometry.

### *In situ* Hybridization

*In situ* hybridization was carried out as in Thisse and Thisse (2014). Briefly, embryos were raised to the desired stage and fixed in 4% PFA (Kimmel et al., 1995). Fixed embryos were permeabilized with proteinase K at 10 μg/mL and incubated with RNA probes at 70°C (probe synthesis described above). Additionally, full probe sequences are provided in the Supplementary Material. Probes were labeled with anti-DIG AP primary antibody (Roche, Basel, Switzerland) at 4°C overnight and then embryos were stained with NBT/BCIP (Thermo Scientific, Rockford, IL, United States). Stained embryos were mounted in 4% methyl cellulose on glass depression slides and imaged on a Nikon SMZ800 dissecting microscope at 5× using SPOT imaging software version 5.2. Images were uniformly adjusted for brightness and contrast in Adobe Photoshop CS6.

### Histology

For zebrafish histology, embryos were staged as above and fixed in 4% PFA. Embryos were embedded using the JB-4 Embedding Kit (Polysciences, Inc., Warrington, PA, United States) and sectioned at a thickness of 7 μm on a Leica RM2265 microtome. Sections were mounted on slides and stained with hematoxylin and eosin. Stained sections were imaged at 20× on an Olympus IX71 microscope. Images were uniformly adjusted for brightness and contrast in Adobe Photoshop CS6.

### Immunohistochemistry

After morpholino injection, fish were raised to the desired stages and fixed in 4% paraformaldehyde. Embryos were washed in PBS + 0.01% Triton X-100 and permeabilized in ice-cold acetone. Primary antibodies, anti-phospho-Histone H3 (Cell Signaling Technologies, Cat. # 3377S, Danvers, MA, United States) and anti-cleaved-caspase 3 (Cell Signaling Technologies, Cat # 9579S, Danvers, MA, United States) were applied to embryos at 1:1000 and incubated at 4°C overnight. Secondary antibodies, anti-rabbit conjugated to Alexa Fluor 555 (Cell Signaling Technologies, Cat. # 4413S, Danvers, MA, United States) were also added at 1:1000 and then incubated at room temperature for 2 h. 18 hpf embryos did not require acetone permeabilization and were mounted in 4% methyl cellulose and imaged wholemount at 10x on an Olympus IX71 microscope. Labeled cells were counted using the ImageJ Cell Counter tool and eye field area was measured using SPOT imaging software version 5.2. After immunostaining, 24 h through 72 h post fertilization embryos were embedded and sectioned as for histology. Sections were counterstained with Vectashield with DAPI (Vector Labs, Burlingame, CA, United States) and imaged at 20× on a Nikon Ti confocal microscope. For the sections, labeled and total cells were counted using the ImageJ Cell Counter tool. All images were compiled and adjusted for brightness and contrast in Adobe Photoshop CS6.

### FLOW Cytometry

After injection, *Tg(Rx3:GFP)* embryos were raised to the 18 somite stage and screened for GFP expression. GFP+ embryos were washed in PBS and dissociated by being pressed through a 70 μm filter. Cells were fixed in Zinc Buffer (0.05% CaAc, 0.5% ZnCl_2_, 0.5% ZnAc, in 1M Tris-HCl) at 4°C overnight (Jensen et al., 2010). Fixed cells were stained with 3 μM DAPI in PBS with 0.1% Triton X-100. Flow cytometry analysis was performed on BD LSRII equipped with a 405 nm laser for excitation of DAPI, and signal detected using a 450/50 BP filter. Unstained cells were used to set up the instrument. Cytometer optimization and calibration were performed as recommended by standard guidelines (Wang and Hoffman, 2017). Fifteen thousand singlet (based on FSC-A vs. FSC-H) events were recorded. Data were acquired using BD FACSDiva software v 8.0.1 and analyzed with FlowJo software v 10.5.3, using similar univariate cell cycle model for all samples (FlowJo, LLC, Ashland, OR, United States).

### Statistical Analysis

All statistical analyses were performed using Graphpad Prism 7. Graphs present all data points and, in most cases, the mean and standard error of the mean (SEM). Tests used are indicated in results but include: ordinary one-way ANOVA followed by Tukey’s multiple comparisons test; two-sided, unpaired *t*-tests; and two-sided, paired *t*-tests.

## Results

### Zebrafish *hars* Is Most Highly Expressed in the Developing Nervous System

**FIGURE 1:**
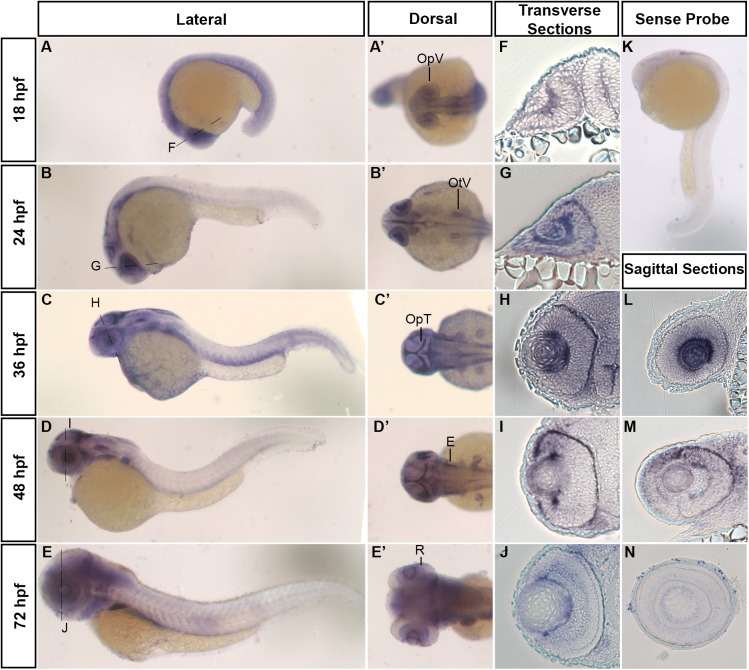
Developmental mRNA expression patterns of zebrafish *hars*. **(A–E)** Lateral and **(A’–E’)** dorsal views of zebrafish embryos at indicated stages, showing *hars* expression. **(F–J)** Transverse sections through the retina (at the level of the dashed lines in **A–E**). **(K)** Lateral view of *hars* sense probe. **(L–N)** Sagittal sections through the retina. OpV, Optic Vesicle; OtV, Otic Vesicle; OpT, Optic Tectum; E, Ear; R, Retina.

We began investigating the role of *hars* in zebrafish development, by asking where and when the gene was most highly expressed. Using *in situ* hybridization, we found that *hars* was most strongly expressed in regions of the developing nervous system (Figure 1A–E’). At 18 h post fertilization (hpf), expression is fairly ubiquitous throughout the embryo but we noted strong expression in the optic vesicles (Figure 1A,A’). At 24, 36, and 48 hpf, there is higher expression in the developing eye, ear, and optic tectum (Figure 1B–D’). At 72 hpf, expression is weaker, however, still found in the retina, ear, and throughout the brain (Figure 1E,E’). Transverse and sagittal sections through the eye further show *hars* expression in areas associated with proliferation (Figure 1F–J, L–N).

In line with these expression patterns, a search through *in situ* hybridization data for mouse *Hars* show similar patterns, with striking expression in the early retina before neurons have started differentiating (Diez-Roux et al., 2011). Additionally, our zebrafish *hars* expression patterns are also similar to the expression of other zebrafish ARS genes, suggesting that this pattern is not exclusive to *hars* (Fukui et al., 2009; Cao et al., 2016; Castranova et al., 2016; Kopajtich et al., 2016; Ni and Luo, 2018; Wang et al., 2018). Overall, these patterns support the idea that *ARS* are uniquely important for the generation of neuronal cells.

### Nervous System Development Is Most Sensitive to Reduced *hars* Expression

**FIGURE 2:**
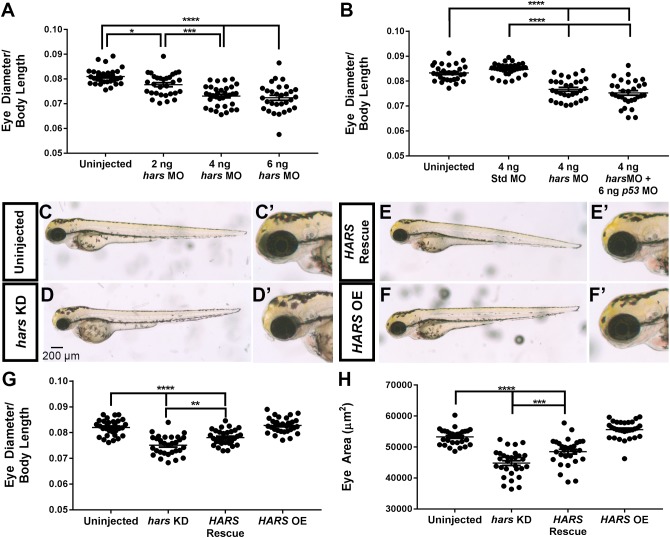
*hars* knock-down decreases eye size. **(A,B)** Quantification of eye diameter relative to body length with **(A)** increasing doses of *hars* morpholino and **(B)** standard morpholino and *p53* morpholino controls. **(C–F’)** Brightfield images of 72 hpf embryos. **(C,C’)** Uninjected, **(D,D’)** *hars* MO, **(E,E’)** *hars* MO plus human *HARS* mRNA, and **(F,F’)** human *HARS* mRNA alone. **(G,H)** Quantification of **(G)** eye diameter relative to body length and **(H)** eye area of rescue experiment. Ordinary one-way ANOVA followed by Tukey’s multiple comparisons test was performed (*n* > = 30 ^∗^ = 0.05, ^∗∗^ = 0.01, ^∗∗∗^ = 0.001, ^∗∗∗∗^ = < 0.001); the error bars indicate mean and SEM.

Based on the *hars* expression profile, we set out to assess how neuronal development is affected by a reduction in *hars* expression. Zebrafish (and the other members of the teleost lineage) are unique among vertebrates in that they have a single *hars* gene that codes for both a mitochondrial and a cytoplasmic enzyme via alternative splicing and translation start sites (humans and other vertebrates have two separate genes) (Waldron et al., 2017). The two transcripts vary at their 5′ end, so to avoid disrupting expression of mitochondrial proteins, we used a knock-down approach designed to block translation of the cytoplasmic *hars* transcript but not the mitochondrial. We first looked at the effect of knocking down *hars* on the development of the entire zebrafish by injecting fish at the one-cell stage and letting them grow for 72 h under normal rearing conditions. Initially, *hars* knock-down embryos appear healthy overall, but upon closer inspection we found that compared to body length, their head and eyes are smaller than uninjected siblings in a dose-dependent manner (Figure 2A). Presumably, there is a level of synthetase at which life is no longer viable, and indeed embryos injected with the highest dose of morpholino showed much more severe, whole body defects. However, to address whether neuronal tissues are more sensitive to *hars*, we chose to proceed with the dose that consistently resulted in the small eye phenotype without severe effects to the rest of the embryo. To control for off-target effects of morpholinos, we used a standard control morpholino and show that these fish have no phenotype, indicating the phenotype is specific to the *hars* morpholino (Figure 2B). In addition, to control for non-specific activation of the p53 pathway, we co-injected a T*p53* morpholino with the *hars* morpholino and found that that phenotype is not *Tp53* dependent (Figure 2B). We also generated mRNA for GFP that had its 5′ end replaced with the *hars* morpholino binding sequence or a 5 base pair mismatch version of the sequence. Injection of these mRNA with and without the morpholino showed that the morpholino could abolish GFP expression from the wildtype mRNA, but had little effect on the 5 base pair mismatch version, supporting the specificity of the morpholino (Supplementary Figure S1). Importantly, we were able to partially rescue eye size by co-injecting mRNA for human *HARS*, confirming the specificity of our morpholino (Figure 2C–H). Despite human and zebrafish HARS being highly conserved (77% identity), there are likely differences in tRNA recognition between the two organisms that could account for the partial rescue as opposed to full rescue (Waldron et al., 2017).

**FIGURE 3:**
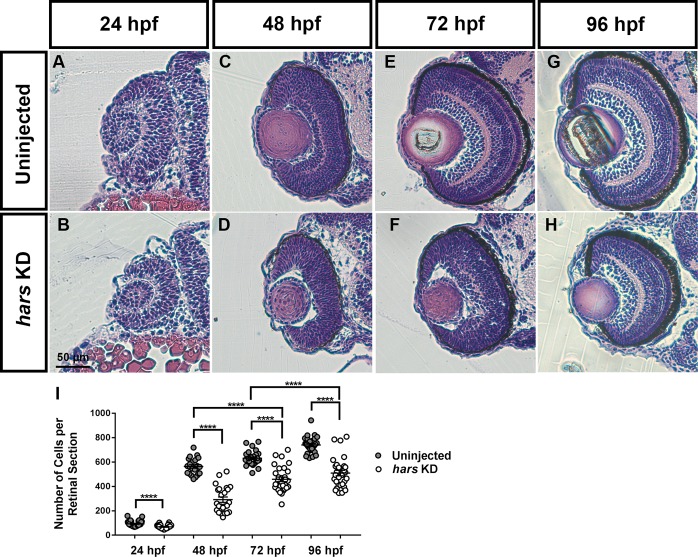
Retinal histology of *hars* knock-down embryos. **(A–H)** Images of 7-micron transverse sections taken through uninjected and *hars* KD embryos at the indicated ages and stained with hematoxylin and eosin. **(I)** Quantification of cell numbers per central retinal section. Cell counts were analyzed by Student’s *t*-test (*n* > = 30 ^∗^ = 0.05, ^∗∗^ = 0.01, ^∗∗∗^ = 0.001, ^∗∗∗∗^ = < 0.001); the error bars indicate mean and SEM.

For concision, we chose to focus our analysis on the retina as it represents a well characterized, accessible subset of the central nervous system (London et al., 2013). We took transverse sections through the retina at 24, 48, 72, and 96 hpf and stained with hematoxylin and eosin to investigate whether there were morphological defects in the retina, and at what developmental stage the phenotype is observed (Figure 3A–H). By counting the number of cells per central retina section, we found that *hars* KD embryos had fewer retinal cells at each age, with the phenotype apparent as early as 24 hpf (Figure 3I). However, there did not appear to be any striking impact on the overall patterning of the retina as all layers are clearly present by 96 hpf.

### *hars* KD Causes Reduced Proliferation and Increased Cell Death in the Eye

**FIGURE 4:**
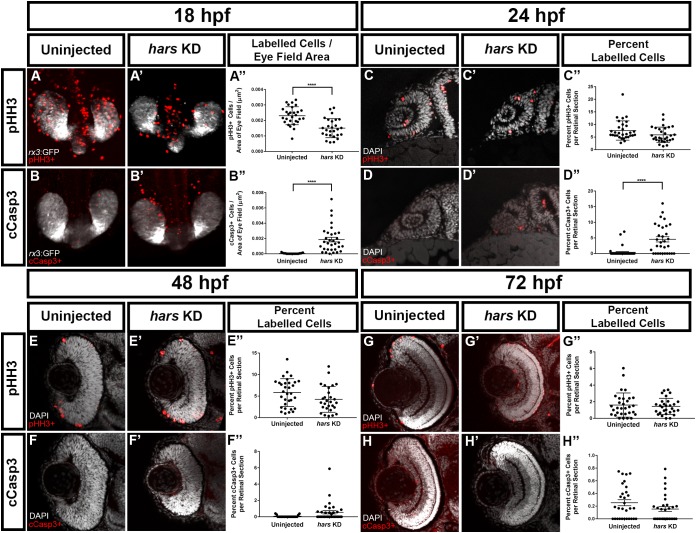
*hars* KD embryos show reduced proliferation as well as increased cell death of retinal progenitor cells **(A–B’)** Confocal images of 18 hpf *Tg(Rx3:GFP)* embryos labeled with **(A,A’)** the mitotic marker phospho-histone H3 (pHH3) and **(B–B”)** the apoptosis marker cleaved-caspase3 (cCasp3). **(C–H)** Single plane confocal images of transverse sections of embryos at **(C–D’)** 24 hpf, **(E–F’)** 48 hpf, and **(G–H’)** 72 hpf labeled with pHH3 or cCasp3 and counterstained with DAPI. **(A”–H”)** Quantification of **(A”–B”)** labeled cells per eye vesicle and **(C”–H”)** labeled cells per retinal section. Student’s *t*-test (*n* > = 30 ^∗^ = 0.05, ^∗∗^ = 0.01, ^∗∗∗^ = 0.001, ^∗∗∗∗^ = < 0.001); error bars indicate mean and SEM.

We next asked whether the reduced cell numbers in the eyes of *hars* KD embryos were due to reduced proliferation or increased cell death. We performed immunohistochemistry for markers of these two processes to determine the extent to which each of them contributes to this phenotype. Because we already observed fewer cells at 24 hpf, we started with an even younger age for these experiments. At 18 hpf, brain derived eye-fields are organizing into an optic cup, which is composed of proliferative retinal progenitor cells. We used a transgenic line that expresses GFP in these cells (*Tg(Rx3:GFP)*) and labeled for either phospho-histone H3 (pHH3) (a marker of cells in M-phase) or cleaved-caspase 3 (cCasp3) (a marker of apoptosis) then imaged the eye-fields dorsally. At this time point, we found that there were significantly fewer proliferating cells as well as more apoptotic cells in the eye fields in *hars* KD embryos (Figure 4A–B”).

Quantifying labeled cells in the intact eye becomes difficult as development progresses and tissues get thicker. For 24 hpf through 72 hpf we performed whole-mount immunohistochemistry as for the 18 hpf embryos, but then took transverse sections through the eyes and quantified the percent of labeled cells in a section from the central retina. At 24 hpf we found that there is no longer a significant reduction in proliferative cells but that there are significantly more apoptotic cells (Figure 4C–D”). By 48 hpf proliferation appears to be equal in both groups, and cell death appears to be only slightly higher in the *hars* KD embryos (Figure 4E–F”). These differences were no longer apparent at 72 hpf (Figure 4G–H”). This data suggests that early decreases in proliferation and increases in cell death contribute to there being fewer retinal progenitor cells available to construct the retina.

### The Amino Acid Starvation Response Is Activated in *hars* KD Embryos

**FIGURE 5:**
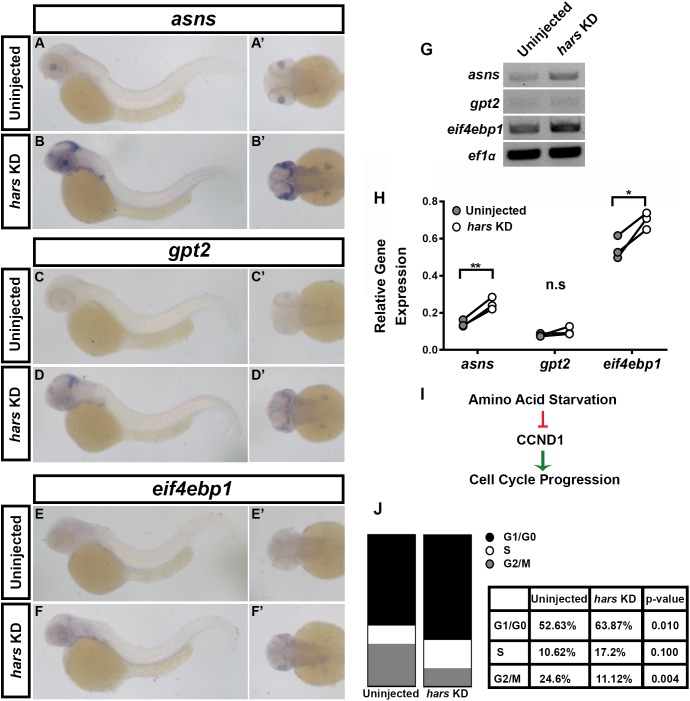
The amino acid starvation response (AASR) is activated in *hars* KD embryos and results in G1-arrest of retinal progenitor cells. **(A–F’)** *In situ* hybridization for three AASR genes in 48 hpf *hars* KD embryos compared to controls. **(G,H)** Semi-quantitative RT-PCR for the AASR genes. **(H)** Quantification of AASR gene expression relative to *ef1α* in control and *hars* KD embryos. **(I)** Schematic showing the relationship between AASR and cell cycle regulation. **(J)** Results of DNA content analysis on GFP+ cells from 18 hpf (*Tg(Rx3:GFP)*) uninjected and *hars* KD embryos. Percentages shown in table are the mean percentages of three replicates. Student’s *t*-test (*n* = 3 ^∗^ = 0.05, ^∗∗^ = 0.01).

Loss of ARS or their function has been shown to lead to an accumulation of uncharged tRNAs, which activate the eif2α kinase GCN2, and ultimately stalls translation which initiates a stress response termed the Amino Acid Starvation Response (AASR) (Harding et al., 2000; Wek et al., 2006). This response drives the transcription of stress-related genes, including *asns*, *gpt2*, and *eif4ebp1* (Sundrud et al., 2009). To test whether this response is activated in *hars* KD embryos we performed *in situ* hybridization and semi-quantitative RT-PCR for these three genes (Figure 5). Both of these measures showed that these three genes were upregulated in *hars* KD embryos, though to varying extents (Figure 5A–H). Interestingly, the *in situs* show that they are upregulated in a tissue specific manner (Figure 5A–F’). In *hars* KD embryos these genes were most strongly expressed in proliferative regions of the nervous system, such as the posterior optic tectum and the region surrounding the lens in the eye (likely the ciliary marginal zone) (Figure 5A–F’). Moreover, these regions are where we saw strong *hars* expression, further supporting the idea that these tissues are particularly sensitive to *hars* KD (Figure 1).

Ultimately, one consequence of the AASR is inhibition of cyclin D1 (CCND1) accumulation (Figure 5I). A lack of CCND1 causes cells to stall in G1 and eventually undergo apoptosis. We used FACS DNA-content analysis to test whether retinal progenitor cells were indeed stalling in G1 in *hars* KD embryos. Analysis of GFP + cells from 18 hpf *Tg*(*Rx3:GFP*) embryos revealed that an average of 52.63% of cells were in G0/G1 in control embryos while an average of 63.8% were in G0/G1 in *hars* KD embryos (Figure 5J). Taken together, these findings support the idea that *hars* KD inhibits cell proliferation by inducing the AASR and that neuronal tissues are particularly sensitive to this stress response.

### *hars* KD Can Be Rescued by Overexpressing *ccnd1*

**FIGURE 6:**
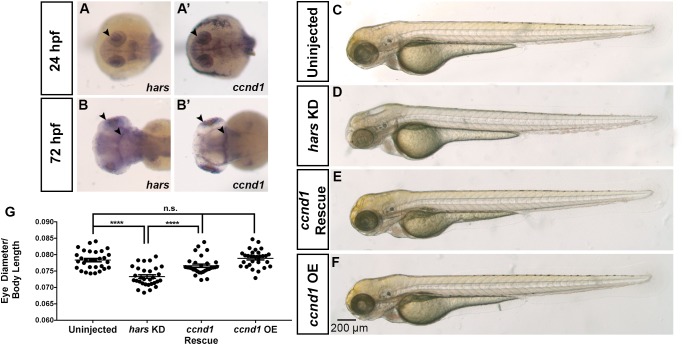
The *hars* KD phenotype can be rescued by overexpression of *ccnd1.* **(A–B’)** mRNA expression of *hars* and *ccnd1* at 24 hpf and 72 hpf. **(C–F)** Brightfield images of **(C)** uninjected embryos, **(D)** embryos injected with *hars* MO, **(E)** embryos co-injected with *hars* MO and zebrafish *ccnd1* mRNA, **(F)** or zebrafish *ccnd1* mRNA alone. **(G)** Quantification of relative eye size at 72 hpf. One-way ANOVA and a multiple comparisons test (*n* > = 30 ^∗^ = 0.05, ^∗∗^ = 0.01, ^∗∗∗^ = 0.001, ^∗∗∗∗^ = < 0.001); error bars indicate mean and SEM.

In addition to the tissue-restricted expression of the stress response genes, we also noticed that *ccnd1* itself shows similar expression patterns to *hars*, further supporting the tissue specific phenotypes observed in *hars* KD embryos (Figure 6A–B’). Another study linked HARS and CCND1 when they identified a temperature-sensitive, cell cycle deficient hamster cell line with a point mutation in the gene for *Hars* that they found was unable to accumulate CCND1 and predictably stalled in G1 (Motomura et al., 1996). Interestingly, they also found that they could rescue the G1 stall by overexpressing *Ccnd1* mRNA. To test this finding in our system, we co-injected mRNA for zebrafish *ccnd1* into *hars* KD embryos and measured relative eye sizes at 72 hpf as in Figure 2. Surprisingly, the overexpression of CCND1 was able to fully rescue the eye size phenotype in the *hars* KD embryos (Figure 6C–G). These results combined with the AASR results indicate that accumulation of CCND1 is indirectly dependent on HARS function.

### Other Neuronal Cell Types Are Affected by *hars* KD

**FIGURE 7:**
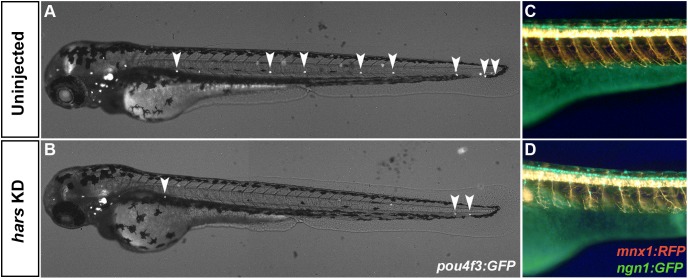
Other neuronal cell populations are affected by *hars* KD. **(A,B)** Combined brightfield and fluorescence composite images of *Tg(pou4f3:GFP)* embryos at 72 hpf labeling neuromasts. **(C,D)** Fluorescence images from the trunk of [*Tg(ngn1:gfp)*); *Tg(mnx1:rfp)* embryos at 72 hpf labeling motor and sensory neurons.

Finally, we chose to look at the effect that *hars* KD had on other neuronal cell types in the zebrafish. Because two of the diseases associated with human *HARS* mutations affect the peripheral nervous system and auditory system, we utilized three transgenic lines that allowed us to look at the sensory neurons, motor neurons, and sensory hair cells. *hars* knock-down in *Tg(pou4f3:GFP)* embryos caused a severe reduction in the number of sensory hair cells seen in the lateral line at 72 hpf (Figure 7A,B). We also noticed axonal branching abnormalities in sensory and motor neuron development when *hars* was knocked down in *Tg(ngn1:GFP);Tg(mnx1:mCherry)* embryos (Figure 7C,D). These results indicate that a reduction in neuronal cell numbers is not unique to the retina and that other neuronal progenitor cell populations are also sensitive to *hars* knock-down.

## Discussion

Over the past 15 years, mutations in genes for ARS have been associated with a myriad of tissue-specific human disorders, forcing us to reexamine our understanding of ARS biology (Antonellis et al., 2003; Meyer-Schuman and Antonellis, 2017). The ubiquitous nature of ARS has meant that specific roles in development have been largely overlooked. In our study, we have revealed an aspect of how one ARS family member, HARS, influences the development of an organism. As many of the ARS mutations, including all of the currently known HARS mutations, appear to mainly affect nervous system development and function, we chose to focus on neuronal development. We show that the zebrafish nervous system, as exemplified by the retina, has a higher demand for HARS and is preferentially sensitive to the cellular stress that is caused by a reduction in HARS expression.

Our results indicate that highly proliferative cells suffer the most severe defects as a result of knocking down HARS expression. However, much of early development is dependent on proliferation, so why would we still see tissue specific defects? This could be due to the method and timing of HARS knock down in the zebrafish. Though we do not directly show that HARS or histidylated-tRNA is maternally deposited, we can assume that one or the other is, since zygotic translation could not occur otherwise. The morpholino used in this study only blocks translation of HARS transcripts, such that maternally provided protein or tRNAs would be available to allow the embryo to make it through the first stages of life. As this source is diluted through many rounds of cell division or used up in the translation of countless proteins, cells dividing later in development that depend on newly made HARS might experience a deficit in protein synthesis capacity. The absence of HARS would promote the accumulation of de-acylated tRNAs in these older cells, thereby activating the eIF2α kinase, GCN2 (Sood et al., 2000). Phosphorylation of eIF2α results in translation attenuation and promotes proteasomal degradation of CCND1, causing proliferative cells to stall in G1 (Hamanaka et al., 2005). When this response is prolonged, the cells can then undergo apoptosis (Wek et al., 2006). We see evidence of both of these outcomes in *hars* KD zebrafish. Furthermore, a previous study had shown that cells containing a temperature-sensitive mutation in Hars also arrest in G1 and fail to accumulate CCND1 when exposed to a high temperature (Motomura et al., 1996). Interestingly, this phenotype could be suppressed by supplementation with high levels of histidine.

Several zebrafish lines with mutations in ARS have been reported. While many of these lines have not been thoroughly characterized, or have not been characterized in the context of nervous system development, all show a gross phenotype similar to our HARS knock-down embryos (Amsterdam et al., 2004; Zhang et al., 2014; Cao et al., 2016; Castranova et al., 2016; Kopajtich et al., 2016; Ni and Luo, 2018). The list of mutants includes a recently published HARS mutant, which exhibits the same small eye phenotype seen in the HARS knock-down (Ni and Luo, 2018). Those mutants that have been analyzed for nervous system defects also show fewer cell numbers in the central nervous system and increased apoptosis, albeit at later ages than what we have observed (Zhang et al., 2014). The phenotypic similarity between the various ARS mutants and ARS knock-down suggest that the neurological phenotypes may result from a shared mechanism, likely tissue specific induction of the AASR.

Many other “housekeeping” proteins have been associated with tissue-specific disorders. One example, the craniofacial disorder, Treacher-Collins Syndrome, can be caused by mutations in components of ribosomal biogenesis and rRNA transcription (Calo et al., 2018). This study reveals differential sensitivity to p53 activity in neural crest cells, which make these cells more susceptible to p53-induced apoptosis and that these differences explain the tissue-specific defects seen in the disease (Calo et al., 2018). In another example, a protein involved in tRNA splicing has been found to cause p53-dependent neurodegeneration in both humans and zebrafish (Schaffer et al., 2014). In our study, we see cell-type specific susceptibility to the AASR, further supporting the idea that specific cells have varying susceptibility to different stressors. However, the reason for this intrinsic variability in stress responses and how other cell types buffer against this stress is still unknown.

Organism-level experiments such as these have revealed that not all cells respond to stressors equally. The tissue specific responses help to explain how mutations in an ARS could cause a deafness-blindness disorder or microcephaly, while leaving the rest of the individual relatively unscathed. As we learn more about the intrinsic differences among cell types we may finally understand why this variation exists and how to counter act it in disease.

## Ethics Statement

This study was carried out in accordance with the recommendations of University of Vermont Institutional Animal Care and Use Committee. The protocol was approved by the University of Vermont Institutional Animal Care and Use Committee Protocol Number: 14-053.

## Author Contributions

AW and CW performed the experiments and analyzed the results. AW designed the experiments and wrote the manuscript. AE provided direction in experimental design, data analysis, general intellectual input, resources, and manuscript editing. CF provided intellectual input, manuscript editing, and resources.

## Conflict of Interest Statement

The authors declare that the research was conducted in the absence of any commercial or financial relationships that could be construed as a potential conflict of interest.
