# Supplementary material for: Knock-Down of Histidyl-tRNA Synthetase Causes Cell Cycle Arrest and Apoptosis of Neuronal Progenitor Cells in vivo
Source: Front Cell Dev Biol. 2019 Apr 26;7:67. doi: 10.3389/fcell.2019.00067 (PMC6524715; doi:10.3389/fcell.2019.00067)
Supplement: Supplementary file 1 [file Data_Sheet_1.PDF]

Supplementary Material

<u>*Full* in situ *probe sequences*</u>

asns (NM_201163)

CCAATGGAAAAAGTCTGAATGGGATATGGAAGCTTCTCTTCTTTGGCCAGTTTCACA
AGCAAAGCAGCAACCAGACTTGAGTCGAGACCACCTGAGAGAAGGCAACCGATTCT
TCTGTGAGCCATCAAGCGTTTCCTTACTGCATCCTCAAACAGGATCCTAATGTTGCTC
TTGACTGTCTCCAATTCGAAGTCTGTGCCCAGTCCCTCGAGTTTGTTAAAGTCAGCAT
GTTTGGGTTTGTCTGTGCAGCAGTGAAAGCGATCCATCTGAACAGACTCCACTTTCC
CATTGAGCTTCAAGTCAAACACCTCAAAGTGTCCCGGTGGAAAAGCAGTGATCTTTG
CAGTGGGCATGGAGTGTTTGATTTGTGTTAGACCTTTGCCTTCAGAACAGACAGCTA
GAAAACCATCATCAGTCAGCATTCTGAATAGCGGTCTCACGCCGTATGTATCTCTTC
CCAAGTGAACCTTTCTGTTAGCTGTGTCTAAT

eif4ebp1 (NM_199645)

CAGACGAGTCATACTCAACGATGCGGCCCATCTGCCTCATGATTACTCGACAACACC
CGGGGGGACCCTTTTTAGCACGACTCCTGGAGGCACCCGGATCATCTATGACCGAA
AGTTCCTGCTGGACTGTCGCAGCTCTCCATTGGCCCGCACACCGCCCTGCTGCCTGC
CTGACATTCCTGGGGTGACCAGTCCTCCTTCAGTCACCGTAAATAATGAAAAGGCCT
ACCCTAAACCCACTGTCAATAATAACAGCATCAGCCCCCCGGTGGACAAGAGCACA
GGGGAGGATGCCCAGTTTGAAATGGACATCTAACTACAGTAGAGGAACTGCTACAC
GAAGGAGACGACGACTGTAATGAATATTACTGTGATGATAAAGATGATTACTGTTCC
CAGAGAGGATAAATGCACAGAAGACTTCTCCTTCCATGTCATCTGTCTTGTTTTTTTT
TCTTGTTTTTTTTTTTTTTGTCATTCATTTACTGTTCAAATGATATTTTCCTT

gpt2 (NM_001098757)

GGTTTCTGGGAAAGACTCTTCTCGGACTGGTGTAATGATCCCCATTCCTCAGTACCC
GCTTTACTCTGCCGCAATCTCAGAGATGGATGCTGTTCAGGTCAACTACTACTTGGA
TGAGGACAACTGCTGGGCCCTGGACATCAATGAACTTCACAGAGCCTATCAGGCTG
CCAAACAGCACTGCCAGCCCCGAGTCATCTGCATCATTAACCCTGGCAATCCTACGG
GTCAGGTTCAGAGTAAAAAATGCATTGAAGAAGTCTTGCACTTTGCTTATGAGGAGA
ACCTCTTTGTGATGTCAGATGAGGTGTATCAGGACAATGTGTATGCTCCAGATTGTC
AGTTCCACTCCTTTAAGAAGGTGCTATACGAGATGGGTCCTGAGTACTACAACAGTG
TGGAGCTCGCTTCTTTCCATTCCACTTCCAAAGGCTACACAGGAGAGTGTGGCTTCA
GAGGAGGTTATATGGAGGTGATCAACATGGATCCTGAGGTCAAGGCGCAGCTGGTG
AAGCTCTTATCC

hars (NM_001302262)

CAAAAGTGAGAAAGCGAGCAAAGAACAGATTGATGAAGAAGTGGCCAGACTGTTG
CAGCTCAAAGCTCAGCTGGGAGGAGACGAAGGAAAACATGTATTTGTTCTCAAAAC

AGCCAAGGGGACCAGAGACTACAACCCCAAACAGATGGCTATACGAGAGAAGGTTT
TCAACATCATCATCAACTGCTTTAAACGCCATGGTGCCGAAACCATCGATTCCCCCG
TCTTTGAGCTGAAGGAAACGTTGACGGGGAAGTATGGAGAAGACTCCAAACTCATC
TATGATCTGAAGGACCAGGGGGGAGAACTTCTGTCTCTGAGATACGACCTCACTGTA
CCATTTGCTCGCTATCTCGCAATGAACAAAATCACCAACATCAAGCGCTACCACATT
GCTAAAGTGTACCGCAGAGACAACCCAGCCATGACCCGGGGCCGATACAGGGAGTT
TTACCAGTGTGATTTCGATATTGCAGGTCAATACGATGCAATGATCCCTGA

ccnd1 (NM_131025)

GCTCGAGGTCTGTGAAGAGCAGAAATGTGAAGAGGAAGTTTTTCCTTTGGCTATGAA
CTACCTGGACAGGTTTTTATCTGTGGAGCCCACCAAAAAAACCAGGTTGCAGCTTTT
AGGAGCAACTTGTATGTTTCTGGCTTCAAAAATGAAAGAGACTGTGCCACTTACAGC
AGAGAAGTTGTGCATATACACGGACAACTCTGTCCGTCCCGGCGAATTATTGCAAAT
GGAACTGCTGGCGCTAAATAAACTGAAGTGGGATCTGGCCTCAGTGACACCACATG
ATTTCATTGAACACTTCCTTGCCAAACTGCCTATACATCAGAGCTCCAAGCAGATAC
TGCGCAAACACGCCCAGACCTTTGTGGCCCTCTGTGCGACAGACGTCAACTTCATCG
CAAGCCCTCCCTCCATGATTGCAGCAGGCAGTGTTGCTGCAGCGGTACAAGGACTGT
ACCTGAAAAGCACCGACAGTTGCCTCTCATCCCAGAACCTCACCAACTTCCTCTCGC
AAGTC

*Figure S1*

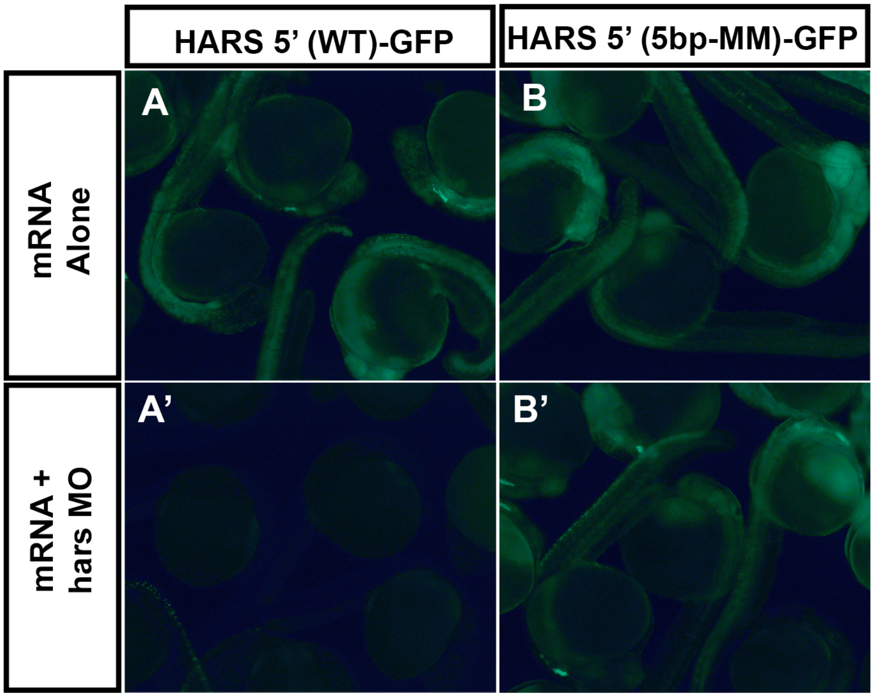

Figure S1. Epifluorescence images of embryos injected with GFP mRNAs which have had the 5' end replaced with either the wildtype *hars* morpholino binding sequence or a 5 base pair mismatch sequence. (A) Embryos injected with GFP mRNA with the wildtype morpholino

binding sequence. (A') GFP expression is abolished when the same mRNA is co-injected with the *hars* MO. (B) Embryos injected with GFP mRNA with the 5 base pair mismatch morpholino binding sequence. (B') GFP expression is retained in embryos co-injected with the 5 base pair mismatch mRNA and the *hars* MO. All images were taken at the same exposure.

Supplementary Methods

We had previously cloned the zebrafish *hars* coding sequence (NM_001302262) into the pExpress vector and the coding sequence for EGFP into the pCS2+ vector. In order to subclone the 5' end of the *hars* coding sequence containing the morpholino binding site into the pCS2+ vector such that it would be in frame with the GFP coding sequence we digested both plasmids with SmaI and NcoI (New England Biolabs, Ipswich, MA). The desired fragments were gel extracted using the QIAquick Gel Extraction Kit (QIAGEN, Hilden, Germany). Fragments were ligated using T4 DNA ligase (New England Biolabs, Ipswich, MA). To generate mRNA from this construct we linearized with NotI (Thermo Fisher Scientific, Waltham, MA) and transcribed *in vitro* with the mMessage mMachine Sp6 Kit (Invitrogen, Carlsbad, CA).

Tg(isl2b:GFP) embryos (gifted by Chi-Bin Chien) were injected with 200 pg of mRNA with or without 4 ng of the *hars* morpholino. Embryos were raised at 28.5˚C to 24 hpf. Pools of embryos were imaged using a 4x objective on an Olympus IX71 microscope. All images were taken using SPOT imaging software version 5.2 at the same exposure level. No post-hoc image adjustments were used.
